# Fecal Indicator Bacteria along Multiple Environmental Transmission Pathways (Water, Hands, Food, Soil, Flies) and Subsequent Child Diarrhea in Rural Bangladesh

**DOI:** 10.1021/acs.est.8b00928

**Published:** 2018-06-14

**Authors:** Amy J. Pickering, Ayse Ercumen, Benjamin F. Arnold, Laura H. Kwong, Sarker Masud Parvez, Mahfuja Alam, Debashis Sen, Sharmin Islam, Craig Kullmann, Claire Chase, Rokeya Ahmed, Leanne Unicomb, John M. Colford, Stephen P. Luby

**Affiliations:** † Woods Institute for the Environment, Stanford University, Stanford, California United States; ‡ Civil and Environmental Engineering, Tufts University, Science and Engineering Complex, 200 College Avenue, Medford, Massachusetts United States; § Division of Epidemiology, School of Public Health, University of California, Berkeley, California United States; ∥ Department of Forestry and Environmental Resources, North Carolina State University, Raleigh, North Carolina United States; ⊥ Civil and Environmental Engineering, Stanford University, Stanford, California United States; # Infectious Disease Division, icddr,b Dhaka 1000, Bangladesh; ¶ Water Global Practice, World Bank, Washington, D.C. 20433, United States; □ Water Global Practice, World Bank, Dhaka 1207, Bangladesh

## Abstract

Enteric pathogens can be transmitted through multiple environmental pathways, yet little is known about the relative contribution of each pathway to diarrhea risk among children. We aimed to identify fecal transmission pathways in the household environment associated with prospectively measured child diarrhea in rural Bangladesh. We measured the presence and levels of *Escherichia coli* in tube wells, stored drinking water, pond water, child hand rinses, courtyard soil, flies, and food in 1843 households. Gastrointestinal symptoms among children ages 0–60 months were recorded concurrently at the time of environmental sample collection and again a median of 6 days later. Incident diarrhea (3 or more loose stools in a 24-h period) was positively associated with the concentration of *E. coli* on child hands measured on the first visit (incidence rate ratio [IRR] = 1.23, 95% CI 1.06, 1.43 for a log_10_ increase), while other pathways were not associated. In cross-sectional analysis, there were no associations between concurrently measured environmental contamination and diarrhea. Our findings suggest higher levels of *E. coli* on child hands are strongly associated with subsequent diarrheal illness rates among children in rural Bangladesh.

**Figure:**
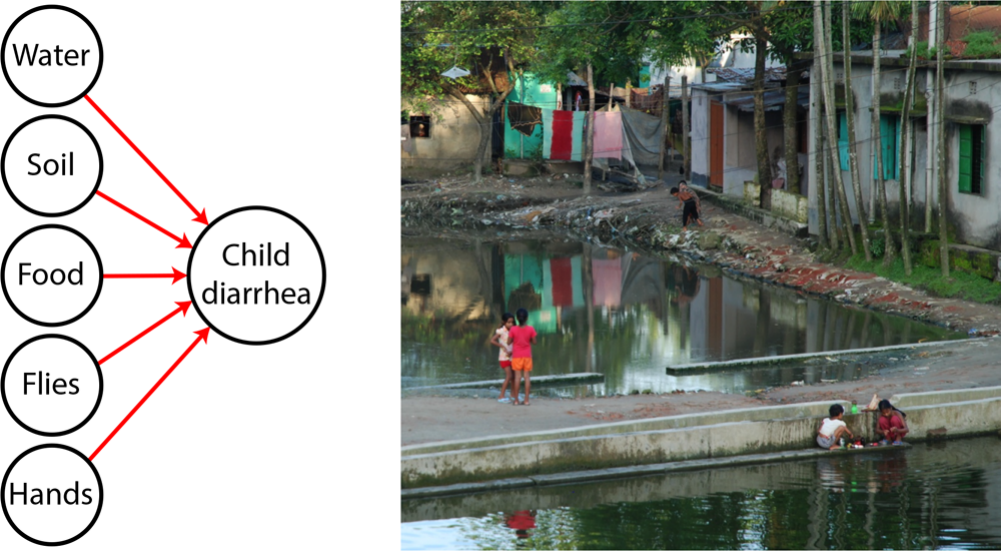

## ■ INTRODUCTION

Diarrhea is a leading cause of global mortality, causing over 1 million deaths in the year 2016.^1^ The morbidity burden of diarrhea is also substantial: in 2010 there were an estimated 1.7 billion episodes of diarrhea.^2^ Diarrheal pathogens are transmitted along multiple environmental pathways, traditionally conceptualized as the “five-Fs”: fluids (water), fingers (hands), food, fields (soil), and flies.^3,4^ Fecal indicator bacteria and some enteric pathogens have been measured in source water, environmental waters, stored drinking water, on child and caregiver hands, in stored food, in soil, and on flies in low-income countries.^5,6^ However, there is limited evidence on which of these pathways are the most important for transmission of diarrhea among young children.^4,7,8^

Additionally, most previous studies have used cross-sectional associations between levels of fecal contamination in the environment and concurrent diarrhea prevalence. For example, the association between fecal contamination levels in stored drinking water and concurrent diarrhea has been extensively studied, with equivocal results.^9,10^ One study in Tanzania found that levels of hand fecal contamination was a stronger predictor of concurrent diarrheal illness within a household than fecal contamination levels in stored drinking water.^11^ Another study in Tanzania found that pathogenic *E. coli* in stored drinking water was associated with a decrease in the odds of concurrent caregiver-reported diarrhea.^12^ Cross-sectional associations are difficult to interpret because the fecal contamination measured in an exposure pathway could have caused the diarrhea, or been caused by the diarrhea, or been influenced by human behaviors elicited in response to the illness (e.g., treatment of water for sick individuals).

The few studies that have prospectively examined fecal contamination along an exposure pathway in a low-income country and diarrhea have focused on drinking water. Luby et al. found that contaminated stored drinking water was associated with diarrhea measured 3–100 days later in rural Bangladesh.^13^ Also in rural Bangladesh, Ercumen et al. reported that prospective measurement yielded a stronger association between *Escherichia coli* in stored drinking water and diarrhea than cross-sectional measurement.^14^ Neither study measured other fecal transmission pathways.

Our objective for this study was to assess fecal contamination along multiple transmission pathways (including drinking water, ambient waters, hands, food, soil, and flies), to better understand their contribution to incident child diarrhea in rural Bangladesh. Diarrheal pathogen transmission pathways are likely heterogeneous across different settings; however, studying the relative risk of diarrhea from exposure to multiple fecal transmission pathways in this setting could provide useful insight into child diarrheal pathogen exposure in similar settings. We also examined how a prospective analysis with incident episodes measured after exposure compared with cross-sectional analysis of environmental fecal contamination and concurrently measured diarrhea prevalence.

## ■ METHODS

**Study Design**. The data collection for this study was nested within the WASH Benefits trial in rural Bangladesh, a multiyear randomized controlled trial of water, sanitation, hygiene and nutrition interventions.^15^ Compounds (extended family groups of 1 or more households sharing a courtyard) were eligible for participation in the WASH Benefits trial if they had at least one pregnant woman in her first or second trimester who did not plan to move in the following 24 months. There were seven arms in the WASH Benefits trial; this substudy only included households from the control, sanitation, and combined water, sanitation, and hygiene (WSH) arms. These arms were selected to allow for analysis of the effect of the sanitation and combined WSH interventions on fecal contamination levels, which will be reported in a separate manuscript. The WASH Benefits trial targeted enrolling 720 households in each intervention arm. Study participants provided written informed consent. Human subjects approval was obtained from the International Centre for Diarrheal Diseases Research, Bangladesh (icddr,b) (PR-11063), University of California, Berkeley (2011–09–3652), and Stanford University (25863).

Data collection for this substudy occurred during the first year of the trial after interventions were delivered and extended from July 2013 through March 2014, spanning both the rainy season (Jul–Oct) and dry season (Nov–Mar). We collected data through two successive visits to each study household. Samples from the household environment were collected during the first visit and analyzed for fecal indicator bacteria levels. Caregiver-reported gastrointestinal illness symptoms in children <5 years were recorded cross-sectionally at this first visit, as well as prospectively at a second household visit. The second visit was targeted to be 4–10 days later; however, due to field logistical constraints, the range of days between the two visits was wider (2–20 days; median 6 days). The spacing between visits was selected to capture symptoms using respondent recall based on typical incubation periods for gastrointestinal pathogens, such as enterotoxigenic *E. coli* (3–4 days), *Salmonella* (2 days), *Shigella* (1–2 days), rotavirus (2 days), and norovirus (1–2 days).^16^

Households that were absent at the time of the field team’s visit were revisited two more times before being marked as loss to follow-up. In households where the pregnant women enrolled into WASH Benefits did not have a live birth or where the study child died after live birth, the team proceeded with the interview and sampling if there was any other child <5 years living in the household. If a household had no other children <5 years, it was considered lost to follow-up.

**Environmental Sampling and Analysis**. We collected source water (tube wells), stored drinking water, pond water, child hand rinse, soil, food, and fly samples from each household. At tube well water sources, field workers removed fabric or other materials attached to the tube well mouth and flushed the tube well by pumping five times.^17^ They collected 250 mL of water directly from the water source using sterile Whirlpak bags (Nasco Modesto, Salida, CA). Field workers asked the respondent to provide a glass of water (in the same way they would fetch it for their <5 children) to obtain a sample of stored drinking water. Field staff asked the respondent to pour the water collected in the glass from the storage container into a sterile Whirlpak bag. If a pond (typically used for bathing or washing dishes or clothes) was present in the compound, a sample was collected from the area where the household reported most commonly accessing the pond by dipping a sterile Whirlpak bag into the pond and collecting 250 mL of pond water. One hand rinse sample was collected from the youngest child in the household. To sample hands, field workers rinsed both of the child’s hands in a Whirlpak bag filled with sterile water.^18^ To collect soil, field workers marked a 30 cm by 30 cm area with a metal stencil (sterilized with ethanol) where the youngest <5 child had most recently played or spent time. The top layer of soil within the stencil was scraped into a sterile Whirlpak bag using a sterile scoop; the sample area was scraped once vertically and once horizontally to collect approximately 50 g of soil from the ground surface. Stored food to be served to children <5 years in the household was collected by asking the respondent to provide a small amount of food in the same manner she fed her child. Food was scooped to fill a 50 mL sterile plastic tube using a sterile spoon attached to the lid of the tube. To capture flies, field workers identified a suitable location in the food preparation area (away from the stove and smoke, under a roof or protected from rain if possible) and horizontally hung three 1.5-foot long strips of nonbaited, sticky fly tape. The fly tape was left in place for 3–6 h to capture flies. Field workers then removed one fly from the center of the strip with the greatest number of flies using metal tweezers that were sterilized with ethanol and placed the fly into a sterile Whirlpak bag.

All samples were preserved on ice and transported to the field laboratory to be processed on the same day, typically within 12 h of collection. Upon arrival at the laboratory, samples were kept on ice until they were processed. Food and soil samples required a homogenization step before processing for fecal indicator bacteria detection. These samples were aliquoted upon arrival in the lab (10 g of food; 20 g of soil) and placed into a sturdy blending bag (BagFilter P, 400 mL, Interscience, Saint Nom, France) with sterile distilled water (100 mL for food; 200 mL for soil). The contents of the blender bag were then homogenized in a laboratory food processer (BagMixer C, Interscience, Saint Nom, France) for 1 min at a specified mixing speed (speed 4 for food and speed 2 for soil). A sterile pipet was used to extract the appropriate volume of the homogenized mixture and further dilute it with distilled water as specified below before processing with the IDEXX Quanti-Tray system. An additional aliquot (5 g) from the original unhomogenized food and soil samples was weighed and placed in a drying oven overnight for determining the sample moisture content to calculate bacterial counts per dry weight of each sample. Fly samples (still encased in the sterile collection bag) were ground with a pestle on a hard surface to homogenize the fly parts. Distilled water (100 mL) was added to the Whirlpak bag containing the pulverized fly. The contents were well mixed and further diluted with distilled water (1 mL of fly mixture was added to 99 mL of distilled water).

Stored water, tube well water, pond water, and hand rinse samples were diluted with distilled water as follows: no dilution for tube well and stored water samples (100 mL of sample processed directly), 2-fold dilution for hand samples (50 mL of sample diluted with 50 mL of distilled water), and 100-fold dilution for pond samples (1 mL of sample diluted with 99 mL of distilled water). All samples were analyzed using the IDEXX Quanti-Tray system with Colilert-18 media (IDEXX Laboratories, Inc., Westbrook, ME) and incubated for 18 h at 44.5 °C.^19^ Ten percent of trays were recounted by the lab supervisor to detect and minimize intercounter variability. One laboratory control (composed of dilution water) per lab technician per day and 2% replicates were processed. Field workers also collected field blanks by pouring sterile water from a sterile bottle into a Whirlpak as if collecting a stored water sample and by opening and massaging a prefilled Whirlpak as if sampling hands.

**Diarrhea Measurement**. At each household visit, field staff recorded the caregiver-reported gastrointestinal symptoms among children <5 years living in the compound. We recorded symptoms for up to three children on the first visit (prioritized by child age, with the youngest sampled first), and we attempted to measure the same children at the second visit. First, the field staff asked if the child had “diarrhea” using the local Bengali term “patla paikana” (“loose stool”). Caregiver-defined diarrhea may be a useful outcome measure since caregivers know the typical frequency and consistency of their children’s bowel movements, and can identify abnormal stool. Field staff then asked if the child had three or more loose or watery stools in a 24-h period, following the World Health Organization definition of diarrhea, and if the child had any blood in their stool (an indicator of more severe diarrhea). Field staff asked illness questions using a recall period of 7-days.^20^ On the second household visit, field staff recorded caregiver-reported diarrhea (both WHO-defined and caregiver-defined) since the previous household visit, as well as in the past 7 days (in order to allow for reporting of the 7-day diarrhea prevalence at both visits). On the second household visit, field staff also recorded if the caregiver reported the child had any rash or bruising in the past 7 days. Rash and bruising were selected as negative control outcomes in the analysis, as they would not be expected to be influenced by levels of fecal contamination in the environment and thus could detect bias in illness reporting.^21^ All household survey responses were recorded electronically using Open Data Kit (ODK) software installed on tablets.

**Statistical Analysis**. We imputed a concentration of zero MPN fecal contamination for fly samples in households where no flies were captured at the food preparation area and zero MPN fecal contamination for households for pond water samples that did not have access to a pond in the compound area (because the absence of flies or ponds would indicate no child exposures via these pathways). MPN counts of fecal indicator bacteria were log_10_ transformed for the analysis; half of the lower detection limit was substituted for samples below the detection limit to calculate the logarithm. Relevant fecal contamination indicators were treated as missing data if the household did not have stored drinking water or food available for sampling, the tube well was temporarily not working or if it was not possible to rinse a child’s hand or collect soil (because these types of missing samples would not indicate lack of child exposure).

We did not prespecify a definition of diarrhea for this analysis, so we estimated all models for each of the following caregiver-reported health outcomes we measured: WHO-defined diarrhea, caregiver-defined diarrhea, and blood in stool. For the prospective analysis, we estimated the incidence rate of these outcomes by dividing the number of new cases since the environmental sampling household visit by the child-days at risk. If a child’s symptoms were not measured on the first household visit, we excluded them from the prospective analysis. For children who did not experience an incident diarrhea episode, we calculated days at risk as the difference between visits. For children with incident diarrhea episodes, we estimated their days at risk assuming onset occurred at midpoint between visits as our data collection instrument did not record the day of symptom onset; assuming disease onset at the midpoint of the follow-up period is unbiased since the interval length in this context is independent of disease status.^22^ In the prospective analysis we quantified the association between *E. coli* levels and diarrhea incidence using the incidence rate ratio (IRR). We modeled binary gastrointestinal illness outcomes as a function of log_10_ MPN in *E. coli* for each pathway using generalized linear models with a log link, a Poisson error structure, an offset for each child’s days at risk, and robust standard errors that treated the study’s geographic clusters as independent units. Each pathway was estimated with a separate model since interactions between pathways could have affected estimates in a multivariable model.^23^ The exponentiated coefficient on the *E. coli* levels estimated the incidence rate ratio (IRR) associated with a 1 – log_10_ increase in *E. coli* MPN. Since rash and bruising were only measured on the second household visit, we conducted the negative control prospective analysis by estimating prevalence ratios. In a sensitivity analysis, we re-estimated results using only data from the control group to remove any potential confounding from intervention.

To compare our prospective analysis with a cross-sectional analysis approach, we repeated the analyses described above using 7-day prevalence of diarrhea measured at the first household visit as the dependent variable (measured simultaneously with the environmental sampling); this analysis used illness prevalence as the outcome measure as incident diarrhea or blood in stool episodes could not be identified at the first household visit without prior knowledge of symptom onset. We estimated the prevalence ratio using a modified Poisson generalized linear model (log link) with robust standard errors.^24,25^

For pathways that were significantly associated with diarrhea in the prospective analysis, we conducted a subgroup analysis by child age to explore if the relationship between fecal contamination and diarrhea incidence was different for children that could have different levels of mobility and contact with each pathway. The age categories were selected based on the US Environmental Protection Agency guidelines^26^ for selecting age groups for monitoring and assessing child exposure to environmental contaminants, as follows: 0–5 months, 6–23 months, and 24 months or older. Since this subgroup analysis was not prespecified, we re-estimated the results using different age cut points as a robustness check. The alternative age categories were selected based on WHO windows of achievement for hands and knees crawling (5–14 months) and walking alone (8–18 months) and were as follows: immobile (0–4 months), rolling, crawling, and learning to walk (5–18 months), and walking well (19–60 months).^27^ We also re-estimated models restricted only to children that provided a hand rinse sample (considering an individual child’s hand contamination might be more closely linked to that individual child’s health). We also conducted subgroup analyses by season (rainy vs dry) to explore if the relationship between environmental contamination in each pathway and subsequent diarrhea was modified by seasonality.

All models controlled for season (rainy vs dry), household wealth (monthly income over or under 6000 BDT [∼$75USD]), and mother’s formal education (0 vs >0 years) as potential confounders of the relationship between environmental contamination and diarrhea, as well as controlled for study arm (control, sanitation, or WSH). The unit of analysis for all models was at the child-level. We did not adjust *p*-values for multiple comparisons.

## ■ RESULTS

A total of 2098 households were eligible for enrollment into this substudy from the control, sanitation, and combined WSH arms of the WASH Benefits trial. Of these, we successfully enrolled 1843 households, with 255 households (12%) lost to follow-up due to stillbirth, miscarriage, abortion, or death of children in the target age range (7.4%), short-term or long-term relocation (3.4%), or refusal (1%). A total of 2430 children <5 years were enrolled into the substudy at the first household visit and are included in the cross-sectional analysis. On the second household visit, 2200 (90%) of these children were successfully measured and included in the prospective analysis. WHO-defined diarrhea 7-day prevalence was 17.8% at the first household visit and 16.9% at the second visit; 7-day prevalence of caregiver-defined diarrhea was 11.9% at the first visit and 10.9% at the second visit; and 7-day prevalence of blood in stool was 1.3% at the first visit and 1.5% at the second visit. WHO-defined diarrhea and caregiver-defined diarrhea were in agreement for 92% of children at the first household visit and 90% of children at the second household visit. The incidence rate of WHO-defined diarrhea between visits was 18 episodes per 1000 child-days (250 episodes during 13882 total child-days at risk); the incidence rate of caregiver-defined diarrhea was 13 episodes per 1000 child-days (192 episodes during 14877 total child-days at risk); and the incidence rate of blood in stool was 3 episodes per 1000 child-days (46 episodes during 16604 total child-days at risk). At the second household visit, 7-day prevalence of rash was 12.2% and 7-day prevalence of bruising was 4.7%. The average number of days between the first and second household visits was 7.7 days (median 6; range 2, 20; interquartile range [IQR] 6).

Among the enrolled households, a total of 9960 environmental samples were collected along seven environmental transmission pathways. Sample collection was evenly distributed over the rainy season (43% of samples, Jul 2013–Oct 2013), and dry season (57%, Nov 2013–Mar 2014). At least one fly was captured from 34% of households; among households with flies, the median number of flies captured was 2 (range 1, 161). Most (80%) food samples were precooked rice, 17% were rice or wheat porridge, and 3% were other types of food. Field staff observed covers on the stored food 85% of the time. All (100%) of sampled food was reported to have been prepared in the home (not purchased outside). Half (49%) of households had access to a pond in their compound. Fecal contamination was prevalent in all seven pathways (Table 1; see Ercumen et al. for additional details on fecal contamination levels and correlation between pathways). *E. coli* was detected in 1% of all lab and field blanks (8 out of 672 total blanks). The Pearson correlation coefficient between replicates was 0.9 (*n* = 233).

**Table 1:** Fecal Indicator Bacteria Levels (Most Probable Number [MPN] *E. coli*) by Pathway^*a*^

	units	*N*	*E. coli*		
			geometric mean^*b*^	IQR	% positive
tube well	100 mL	1676	1	0	24
stored water	100 mL	1627	4	20	58
pond	100 mL	824	5393	14350	98
child hands	2 hands	1772	7	8	40
soil	dry g	1799	117979	938650	94
food	dry g	1650	2	18	59
flies	fly	612	715	9670	54

a Interquartile range (IQR) is the difference between the 25th and 75th percentiles; IQR includes samples with zero E. *coli* detected.

b Geometric mean calculated by including value of 0.5 MPN for samples under the detection limit.

**Figure 1:**
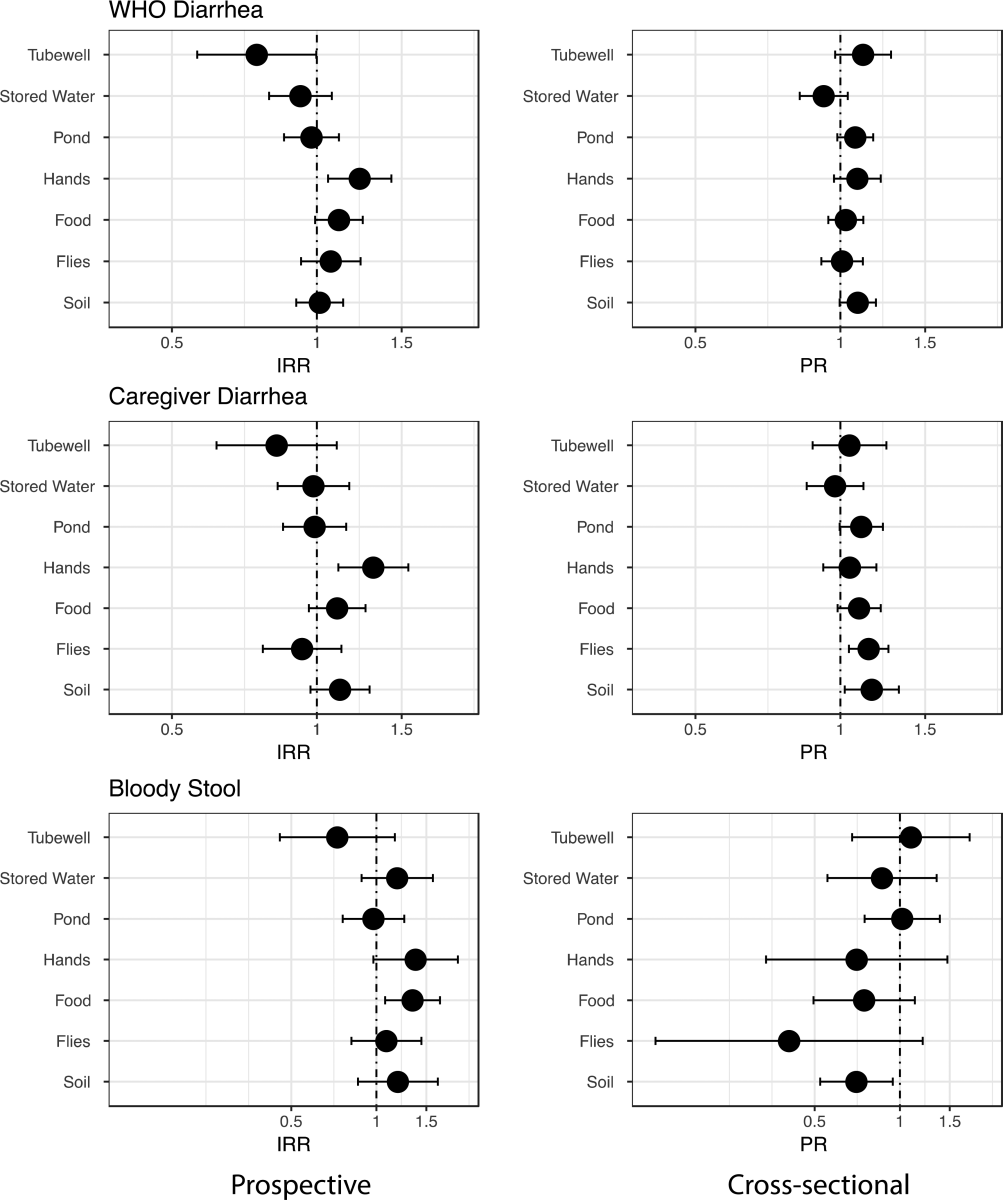
Estimates of World Health Organization (WHO) defined incident diarrhea (3 or more watery stools in 24 h), caregiver-defined incident diarrhea, and incident bloody stool associated with log_10_ MPN *E. coli* in each fecal transmission pathway, prospectively (left column) and concurrently (right column). Incidence rate ratio (IRR) and prevalence ratios (PR) estimated with generalized linear models with a log link, a Poisson error structure, an offset for each child’s days at risk (IRR models only), and robust standard errors; models were adjusted for season, monthly income, mother’s education, and study arm. The axis for bloody stool is on a different scale than the other outcomes to accommodate wider confidence intervals.

**Prospective Analysis**. Estimates of association between environmental contamination and diarrhea were very similar for WHO-defined diarrhea and caregiver-defined diarrhea (Figure 1 and Tables S1 and S2). Higher *E. coli* levels on child hands were significantly associated with both incident WHO-defined and caregiver-defined diarrhea. We estimated the WHO-defined diarrhea incidence rate was 23% higher with each log_10_ increase in *E. coli* on child hands (*n* = 1875 children, IRR = 1.23, 95% CI 1.06, 1.43) and 51% higher if any *E. coli* was detected on child hands (IRR = 1.51, 95% CI 1.17, 1.95; Table S1). Hand contamination was also the only pathway associated with caregiver-defined diarrhea (Figure 1 and Table S2). Restricting the model to include diarrhea data from only those children that had their hands sampled for fecal indicator bacterial levels (instead of all <5 children in the compound) showed the same relationship between hand contamination and incident WHO-defined diarrhea (*n* = 1381 children, IRR 1.23, 95% CI 1.05, 1.44).

Higher *E. coli* levels in food were significantly associated with subsequent blood in stool (Figure 1 and Table S3). We estimated that the incidence rate of bloody stool was 34% higher with each log_10_ increase in *E. coli* in stored food (IRR = 1.34, 95% CI 1.07, 1.68), (Table S3), and the bloody stool incidence rate increased by more than 2-fold when *E. coli* was present in food (IRR = 2.64, 95% CI 1.17, 5.98). *E. coli* presence on hands was also positively associated with bloody stool incidence (IRR = 1.91, 95% CI 1.04, 3.51), while each log_10_ increase of *E. coli* on hands were marginally associated with bloody stool (IRR = 1.37, 95% CI 0.97, 1.94) (Table S3).

The mean prevalence of *E. coli* on hands across child age ranges was: 37% for 0–5 months, 45% for 6–23 months, and 40% for 24–60 months. Subgroup analyses by child age ranges revealed that hand fecal contamination was a statistically significant risk factor for diarrhea among children aged 0–5 months, but not for children aged 6–23 months or 24–60 months (Table 2; results are similar if model is restricted to only those children that provided hand samples (data not shown). Using different age range cut-offs gave similar results; hand contamination was only a significant risk factor for children aged 0–4 months (Table 2).

**Table 2:** Prospective Associations between WHO-Defined (Left) and Caregiver-Defined (Right) Diarrhea and Fecal Indicator Bacteria (log10 MPN *E. coli*) Measurement on Child Hands, Stratified by Child Age Range in Months^*a*^

	WHO-defined diarrhea						caregiver-defined diarrhea				
	age (months)	children (n)	child-days at risk	IRR	95%	CI	children (*n*)	child-days at risk	IRR	95%	CI
EPA categories	0–5	1007	7505	1.29	1.10	1.51	1096	8384	1.44	1.16	1.77
	6–23	424	2672	0.99	0.70	1.40	432	2764	1.04	0.69	1.55
	24–60	444	3700	1.26	0.85	1.85	443	3729	1.22	0.79	1.90
WHO categories	0–4	796	5874	1.37	1.13	1.67	871	6622	1.55	1.24	1.96
	5–18	613	4097	1.10	0.87	1.39	635	4321	1.10	0.84	1.45
	19–60	465	3906	1.23	0.84	1.79	465	3935	1.17	0.77	1.80

a Age categories based on Environmental Protection Agency (EPA) exposure guidelines and World Health Organization (WHO) mobility windows. Incidence rate ratio (IRR) estimated with generalized linear models, adjusted for season, monthly income, mothers’ education, study arm, and child days at risk.

The incidence rate of WHO-defined diarrhea was 14 episodes per 1000 child-days at risk during the rainy season and 22 episodes per 1000 child-days at risk during the dry season; the incidence rate of caregiver-defined diarrhea was 12 episodes per 1000 child-days in the rainy season and 14 episodes per 1000 child-days in the dry season; and the incidence rate of blood in stool was 3 episodes per 1000 child-days in the rainy season and 2 episodes per 1000 child-days in the dry season. During the dry season, the incidence rate of WHO-defined diarrhea was 29% higher with each log_10_ increase in *E. coli* on child hands (IRR = 1.29, 95% CI 1.06, 1.56), while there was no relationship during the rainy season (IRR = 1.14, 95% CI 0.90, 1.46) (Table S6). Similar results were observed for *E. coli* levels on child hands and caregiver-defined diarrhea (IRR when dry = 1.43, 95% CI 1.13, 1.81; IRR when rainy = 1.19, 95% CI 0.94, 1.50; Table S6). During the dry season, the incidence rate of blood in stool was 93% higher with each log_10_ increase in *E. coli* on child hands (IRR = 1.93, 95% CI 1.26, 2.95) and 48% higher with each log_10_ increase in *E. coli* in food (IRR = 1.48, 95% CI 1.16, 1.89); these associations with blood in stool were not present or were attenuated in the rainy season (Table S6). No other pathways were significantly associated with illness outcomes in the rainy or dry season.

*E. coli* levels in all transmission pathways were not significantly associated with our negative control health outcomes (rash and bruising) in the prospective analysis (Table S4). When we restricted the analysis to only include data from the control group, *E. coli* levels on child hands remained statistically significantly associated with subsequent diarrhea by both diarrhea definitions and at similar magnitudes as the full sample (WHO-defined IRR = 1.25; caregiver-defined IRR = 1.26; Table S5). In the control group analysis, *E. coli* levels in food and on hands were no longer statistically significantly associated with incident bloody stool although the point estimates were in the same direction of effect (Table S5).

**Cross-Sectional Analysis**. Simultaneous measurement of child health and environmental fecal indicator bacteria levels did not reveal any statistically significant associations with WHO-defined diarrhea, however the magnitude and direction of the effect estimates were for the most part similar to the effect estimates for caregiver-defined diarrhea (Figure 1 and Tables S1 and S2). We estimated statistically significant and positive associations between 7-day prevalence of caregiver-defined diarrhea and concurrent fly and soil *E. coli* contamination levels (Figure 1, Table S2). In contrast, *E. coli* levels in soil were negatively associated with concurrent bloody stool (Figure 1 and Table S3).

## ■ DISCUSSION

We examined *E. coli* contamination levels along seven different diarrheal illness transmission pathways in the household environment (source water, stored water, pond water, child hands, courtyard soil, complementary food, and flies caught in the compound) as risk factors for incident diarrhea among children under five. Increased *E. coli* levels on child hands predicted incident child diarrhea episodes, while other pathways were not significantly associated. *E. coli* levels in food and on child hands were predictors of a child presenting with bloody stool.

Each log_10_ increase in *E. coli* measured on the hands of the youngest child in the compound was associated with a 23% increase in the incident diarrhea rate among all children <5 years in the compound. The magnitude of this association appears plausible based on two prospective studies in rural Bangladesh that reported each log_10_ increase in *E*. coli measured in stored drinking water increased subsequent diarrhea prevalence by 14% and 50% (these studies did not report incidence estimates).^13,14^ Molecular analysis of a subset of the child hand rinse samples collected in this study detected rotavirus on 6% of child hands (other pathogens were not tested).^28^ Caregiver hands in Tanzania have also been found to carry viral and bacterial diarrheal pathogens, including pathogenic *E. coli*, rotavirus, enterovirus, adenovirus, and norovirus.^12,29^ Our finding could reflect the fact that hands are an intermediary pathway between exposure to other pathways and oral ingestion of fecal contamination; we note that hand *E. coli* contamination levels were significantly correlated to pond and soil fecal contamination in our study site.^30^ Although hand contamination levels could have varied among children in the compound, restricting the model to only include health information from children that had provided a hand sample gave similar results, suggesting hand contamination of one child may represent hand contamination among other children within the same compound. This is consistent with a finding from rural Tanzania that hand contamination levels among children and caregivers were correlated within the same household.^11^

The relationship between hand contamination and incident diarrhea was strongest among children aged 0–5 months old in our study. This finding is consistent with the trend of decreasing hand-mouthing contacts with increasing child age documented in our study population.^31^ In addition, children aged 0–5 months may be more likely to be exclusively breastfed (limiting exposure to contaminated food or water)^32^ and are not yet mobile (limiting exposure to soil and other environmental surfaces).

*E. coli* levels in food and presence of *E. coli* on child hands were predictors of incident bloody stool, while only hand *E*. coli levels were predictive of incident diarrhea. Bloody stool is caused by specific pathogens that could have different transmission pathways in the household environment than other diarrheal pathogens.^33^ For example, S*higella, Campylobacter*, and *Salmonella* can cause bloody stool and are commonly transmitted through food.^16^ Caregivers may also recall bloody stool more accurately than loose stool.^34^ The associations we report between *E. coli* and bloody stool should be interpreted with the caveat that bloody stool was a rare outcome in this study, and underpowered analyses reduce the likelihood that detected effects are reflective of true effects.

One potential causal mechanism for child hand contamination is contact with soil, including direct contact or contact with soiled surfaces. A typical soil sample in our study had >100 000 MPN *E. coli* and >200 000 MPN fecal coliform per dry gram.^30^ A subset of the soil samples in this study was analyzed for molecular markers of fecal contamination; over two-thirds (331/497 samples) of these had ruminant fecal contamination detected and one-third (165/497 samples) had avian fecal contamination detected (lower prevalences were detected in stored water and on hands).^28^ Children 6–23 months old had the highest levels of *E. coli* on their hands (mean log *E. coli* 0.90, SD 0.84), an age range during which children spend significant time crawling on the ground.^27^ Levels of fecal bacteria on child hands could be representative of fecal contamination levels in the broader household environment.

All identified prospective associations between increased *E. coli* levels in the household environment and subsequent gastrointestinal illness were stronger in the dry season and were attenuated or not apparent in the rainy season. All pathways had increased levels of *E. coli* during the rainy season compared to the dry season.^30^ It is possible that *E. coli* contamination on child hands and in food might be dominant diarrheal transmission pathways in the dry season, when overall fecal bacteria levels in the domestic environment are lower. The seasonal differences in our results are consistent with previous evidence that weather can affect the transmission of diarrheal pathogens. Increased temperatures have been associated with increased diarrhea risk,^35–37^ while others have documented increased diarrhea after heavy rains and during dry periods.^38,39^

Soil *E. coli* levels and fly *E. coli* levels were positively associated with caregiver-defined diarrhea in the cross-sectional analysis but not WHO-defined diarrhea (Figure 1 and Table S2). The positive associations could be caused by child diarrhea leading to increased contamination in the domestic environment (reverse causality). For example, child diarrhea could theoretically increase fecal contamination levels in soil if the diarrheal feces or anal wash water were not properly captured and disposed in the latrine, and flies can accumulate fecal indicator bacteria from exposed diarrheal feces. In contrast, increasing fecal indicator bacteria contamination levels on flies and soil were identified as protective for bloody stool in the cross-sectional analysis. One hypothesis to explain this result is that caregivers may be more likely to engage in effective hygiene behaviors (e.g., disposing of feces safely into the latrine) during a child’s severe illness episode, but not necessarily during a less severe diarrhea episode without blood.^40^ The differences in results between prospective and cross-sectional analyses suggest studies should not interpret cross-sectional associations between fecal indicator bacteria contamination levels and diarrhea to indicate a causal relationship of environmental contamination leading to child illness.

The two definitions of child diarrhea used in this study gave similar results in our analysis (Figure 1). Both definitions are widely used in studies of diarrheal disease.^41^ One advantage of caregiver-defined diarrhea is that caregivers are familiar with their child’s normal bowel movements; for example, a healthy breastfeeding child with 3 or more loose stools in 24 h would be classified as having diarrhea by the WHO definition but may not by the caregiver. A disadvantage is that caregivers could fail to recognize clinical diarrhea if symptoms are not severe.^42^ We cannot rule out that our caregiver-reported diarrhea outcomes were subject to bias in this analysis. Caregivers receiving a health intervention might be less likely to report that their child has diarrhea.^43^ Caregiver-reported diarrhea could therefore provide overestimates of the associations between fecal indicator bacteria and diarrhea if intervention recipients both experience reductions in environmental contamination and underreport diarrhea. However, our research team reports no differences in *E. coli* levels on child hands, soil, food, or flies between the intervention arms and the control group in a separate paper (Ercumen 2018, under review). To address potential reporting bias in intervention arms, we controlled for intervention status in all models presented in this manuscript. Additionally, our results were similar when we restricted the analysis to only the control group (one-third of the sample size) (Table S5).

This study had several limitations. First, we relied on caregiver-reported symptoms of gastrointestinal illness, which could be subject to bias, as discussed above. Indeed, the measures of diarrhea and negative control outcomes were both substantially higher in the subset of children included in this analysis compared with the broader WASH Benefits study population.^44^ The reasons for these differences are not clear, though the children in the present analysis were somewhat younger and were assessed by a different separately trained team. However, caregiver-defined and WHO-defined diarrhea yielded similar associations with measures of environmental contamination in our prospective analysis. No significant associations between *E. coli* and negative control outcomes (rash and bruising) suggests that measurement bias was unlikely to have led to the observed associations between *E. coli* and diarrhea, assuming the negative control symptoms and diarrhea were subject to the same reporting bias. Second, *E. coli* could behave differently in the environment than diarrheal pathogens and thus could be a noisy indicator of exposure.^45^ We measured *E. coli* as an indicator of bacterial fecal contamination, which could represent bacterial diarrhea pathogens better than viral or protozoan diarrhea pathogens. Notably, *Shigella* spp. (which is highly genetically similar to enteroinvasive *E. coli*) was identified as the most attributable pathogen to child diarrhea by the GEMS study in Bangladesh, while rotavirus, Adenovirus 40/41, and *Campylobacter* spp. were also identified as important diarrheal pathogens.^33^ Third, our methods to measure *E. coli* had different detection limits and recovery efficiencies across pathways, which could have influenced their relative significance. For example, low recovery of bacteria from food could have masked the association between food bacteria levels and diarrhea. Our results confirm that *E. coli* measurements in environmental samples (particularly solid media such as soil and food) are highly variable, which necessitates large sample sizes for valid inference. Finally, the results from our study site in rural Bangladesh may not be generalizable to other settings with different climates, diarrheal etiologies, and water and sanitation infrastructure.

Prospective analysis may be necessary to accurately characterize the relationship between exposure to fecal contamination and diarrhea. Our results suggest that high levels of fecal indicator bacteria on child hands is strongly associated with incident diarrheal illness among young children in rural Bangladesh. Further research should be done to confirm this association in additional settings.

## ■ ASSOCIATED CONTENT

### Supporting Information

The Supporting Information is available free of charge on the ACS Publications website at DOI: 10.1021/acs.est.8b00928.

Prospective and cross-sectional associations between *E. coli* contamination along each pathway and World Health Organization (WHO) defined diarrhea; prospective and cross-sectional associations between *E. coli* contamination along each pathway and caregiver-defined diarrhea; prospective and cross-sectional associations between *E. coli* contamination along each pathway and bloody stool; prospective associations between *E. coli* contamination along each pathway and negative control outcomes (rash and bruise); subgroup analysis of associations between *E. coli* contamination along each pathway and illness in the control group only; and subgroup analyses showing associations between illness and *E. coli* contamination along each pathway by season (rainy and dry) (PDF)

## ■ AUTHOR INFORMATION

### ORCID

Amy J. Pickering: 0000-0001-6193-2221

Ayse Ercumen: 0000-0001-6002-1514

### Notes

The authors declare no competing financial interest.
